# Multistate Model Builder (MSMB): a flexible editor for compact biochemical models

**DOI:** 10.1186/1752-0509-8-42

**Published:** 2014-04-04

**Authors:** Alida Palmisano, Stefan Hoops, Layne T Watson, Thomas C Jones Jr, John J Tyson, Clifford A Shaffer

**Affiliations:** 1 Department of Computer Science, Virginia Polytechnic and State University, 2202 Kraft Drive, Blacksburg, VA 24060, USA; 2 Department of Biological Sciences, Virginia Polytechnic and State University, 1405 Perry Street, Blacksburg, VA 24061, USA; 3 Virginia Bioinformatics Institute, 1015 Life Science Circle, Blacksburg, VA 24061, USA; 4 Department of Mathematics, Virginia Polytechnic and State University, 225 Stanger Street, Blacksburg, VA 24061, USA

**Keywords:** Systems biology, Biological networks, Mathematical modeling, Chemical reaction systems, COPASI, SBML, Software, Model editor, Multistate

## Abstract

**Background:**

Building models of molecular regulatory networks is challenging not just because of the intrinsic difficulty of describing complex biological processes. Writing a model is a creative effort that calls for more flexibility and interactive support than offered by many of today’s biochemical model editors. Our model editor MSMB — Multistate Model Builder — supports multistate models created using different modeling styles.

**Results:**

MSMB provides two separate advances on existing network model editors. (1) A simple but powerful syntax is used to describe multistate species. This reduces the number of reactions needed to represent certain molecular systems, thereby reducing the complexity of model creation. (2) Extensive feedback is given during all stages of the model creation process on the existing state of the model. Users may activate error notifications of varying stringency on the fly, and use these messages as a guide toward a consistent, syntactically correct model. MSMB default values and behavior during model manipulation (e.g., when renaming or deleting an element) can be adapted to suit the modeler, thus supporting creativity rather than interfering with it. MSMB’s internal model representation allows saving a model with errors and inconsistencies (e.g., an undefined function argument; a syntactically malformed reaction). A consistent model can be exported to SBML or COPASI formats. We show the effectiveness of MSMB’s multistate syntax through models of the cell cycle and mRNA transcription.

**Conclusions:**

Using multistate reactions reduces the number of reactions need to encode many biochemical network models. This reduces the cognitive load for a given model, thereby making it easier for modelers to build more complex models. The many interactive editing support features provided by MSMB make it easier for modelers to create syntactically valid models, thus speeding model creation. Complete information and the installation package can be found at http://www.copasi.org/SoftwareProjects. MSMB is based on Java and the COPASI API.

## Background

Most scientists think of a file editor as nothing more than a computer application used to produce a formatted file. It can be equipped with functionalities to facilitate the production of the desired format, but editors often force the user to adapt his/her own actions to meet the requirements of the final format. Biochemical reaction modeling is a subtle process that requires a lot of creativity. But most current model editors interfere with creativity by forcing the user to focus too much attention on the mechanics of expressing the model in terms acceptable to the editing software. For example, many editors require that the addition of elements to the model follow a specific order, or else fixed default elements are imposed to make the current model syntactically valid. Similar restrictions occur when renaming or deleting elements. The problem is that many editors cannot temporarily represent inconsistency in models, and so they force the user to conform his or her actions to always maintain consistency. In contrast our philosophy is that the editor should focus on user support throughout the entire modeling process. For the publishing industry, “editing” originally meant a process that “*begins with the author’s idea for the work itself, continuing as a collaboration between the author and the editor as the work is created. As such, editing is a practice that includes creative skills, human relations, and a precise set of methods.*” [1].

Today’s biochemical model editors focus more on providing a *precise set of methods*, often forgetting about *human relations* and *creative skills*. In implementing our new model editor, MSMB, we focus on providing ways to adapt the tool response according to personal preferences. For example, the modeler can decide how a delete action propagates through model structure, or which default values to use for newly created species. MSMB supports each step of model creation, providing context-dependent autocompletion and hints that help the modeler to follow a flexible path towards a final coherent and consistent SBML model. MSMB collaborates with the modeler by providing visual clues about what is wrong or incomplete. We take this approach because writing a model is a creative effort in which the user should be allowed to make temporary “mistakes” and recover from them at a time that is convenient rather than having to focus on correct syntax at all times. Most of the existing biochemical editors offer some of the featured just described (e.g., autocompletion, parsing errors, different color layouts) however at the best of our knowledge none of them implements all those features together, thus not properly supporting some of the possible modeling paths.

Representational capabilities of the modeling language have a major effect on the ease or even the ability to create complex biochemical models. A powerful language can concisely express models at a higher level of abstraction, making models shorter to represent and (more importantly) easier to understand. MSMB provides a powerful syntax for representing multistate modeling constructs, an important concept that reduces the number of reactions needed to represent many molecular systems.

### Related work

Currently there are many tools that can be used to edit biochemical reaction models in the form of SBML files (e.g., COPASI [2], CellDesigner [3], Virtual Cell [4]), but most of them enforce strict coherence of the entire model at all times. CellDesigner forces the user to define a species before any reaction involving that species can be defined, which might seem sensible but is a cumbersome requirement. COPASI on the other hand creates all missing species automatically, which can present a problem if the user makes a typo. MSMB combines the two approaches as it allows creation of reactions without prior existence of species, but can be configured to warn the user when species are automatically inserted.

We know of three tools that support multistate modeling in some form. Antimony [5] is a model definition language that allows users to specify parts of the model in a scripting-like language, and then combine these parts to create more complex models. While the ideas of modularity and a human-oriented language are key for Antimony, the editor for the language supports only import/export to SBML and basic text editing (i.e., no autocompletion capabilities, nor on-the-fly parsing/validation, nor guidance for the user about what is wrong in the model).

BioNetGen [6,7] is widely used and supports the concept of multistate species. BioNetGen allows the user to assign “sites” to a species, and each site can have more than one state. However, in the BioNetGen language the states have no relationship with each other. This means that each single transition between states has to be explicitly defined by the user in a separate reaction. This makes some model changes (e.g., adding more states to a site) a manual, error-prone process done through copy-paste. This is in contrast with our compact syntax that includes a state order relation and compact predefined operators for state transitions. For example, in our syntax it is possible to define reactions that change the state of a species from the current one to its “successor” as in this single (parametric) reaction:

(1) $$\begin{array}{lll} \;\text{Reaction definition:} & \;\text{Cdh1(p\{0:maxP-1\})}+\text{ClbM}\; & \;\\ & \text{->}\;\text{Cdh}1(\text{succ}(p))+\text{ClbM},\; \end{array}$$

(2) $$\begin{array}{l} \text{Species definition: Cdh1(p\{0:maxP\}).} \end{array}$$

Equation (2) encodes a species (Cdh1), with one site (p) with consecutive integer states (0 to maxP). Equation (1) encodes a single reaction that represents the set of reactions where each state in the range 0 to maxP-1 is connected to its respective successor state (1 to maxP). Changing the number of sites in this model would require only a change to the numerical value of maxP. Section ‘MSMB multistate syntax’ provides detailed explanations of the different parts of our syntax and more complete examples.

BioNetGen’s editor (RuleBender [8]) offers basic import/export to SBML and a visual representation of the model with complex interconnected entities. However they do not offer autocompletion support in text editing mode or messages to guide the user toward building a valid model.

Simmune [9] is a suite of software tools to define molecules through specification of their submolecular components (i.e., domains or binding sites) using a graphical representation. Molecules form complexes through reactions such as association, dissociation, and transformations. Similarly to BioNetGen, Simmune focuses on individual binding interactions and a small number of transitions between different states. It does not support an explicit order between states, so each transition has to be explicitly listed by the user.

Simulators for multistate systems exist for models written using the BioNetGen language and Simmune format. MSMB does not offer a specific simulation environment because it expands the multistate representation into the equivalent non-multistate model as represent by standard SBML. This allows the user to choose any simulation and analysis software that supports the SBML format. MSMB’s goal is to offer a model-building environment that simplifies the maintenance and testing of models, with an emphasis on providing order and structured operators on states, a concept that has not been included in any of the existing tools that deal with multistate modeling.

## Implementation

MSMB is implemented in Java and uses the COPASI API [2]. MSMB runs on all major operating systems, with minimal system requirements. Installers, source code, user manual, and examples can be found at the MSMB website (http://www.copasi.org/SoftwareProjects).

## Results and discussion

### MSMB primary features

**Figure 1:**
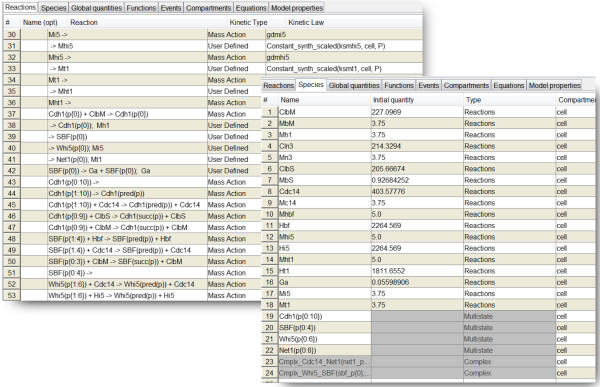
**The MSMB spreadsheet interface.** Each tab contains one part of the model definition (Reactions, Species, Global Quantities, etc.). Reactions and species of the model presented in Section ‘Multisite phosphorylation’ are shown.

MSMB’s spreadsheet interface is shown in Figure 1. The main features that differentiate this editor from existing modeling editors are listed next.

#### 

##### 

**Support for multistate species.** A multistate species is an entity with a defined set of variables (called sites), each associated with an ordered list of states that describes different forms for the same conceptual species. For example, a protein might have multiple phosphorylation sites, each with many distinguishable states of phosphorylation (but each site might have different numbers of phosphorylation states). Multistate species are involved in multistate reactions, which represent a collection of reactions each driven by the same kinetic law but applied to different states of the species. We introduce a compact syntax with operators (like successor/predecessor) on the site’s states. This compact description for batches of similar reactions reduces the cognitive load associated with maintaining and testing different hypotheses on a given model. Section ‘MSMB multistate syntax’ illustrates details of the syntax on models of cell cycle regulation and mRNA transcription in budding yeast.

For one model of the budding yeast cell cycle [10], the original 59 species and 220 single-state reactions are reduced by 67% using MSMB’s multistate representation. When necessary, the compact form of the syntax can be automatically expanded into single-state reactions in order to allow export of the model to standard SBML format.

##### 

**Figure 2:**
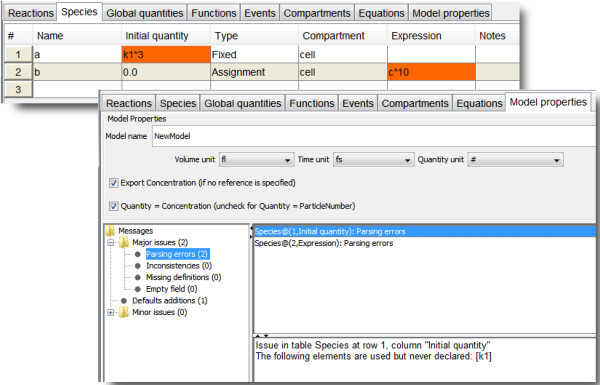
**Warnings and errors are presented to the user in different ways by MSMB.** The table at the top shows that the user defined two species (*a* and *b*) whose expressions involve other elements of the model (*k1* and *c*) not yet defined. MSMB colors the cells referring to undefined elements, and provides error messages (listed in the “Model properties” tab) that explain the errors and warnings in more detail.

**Consistency checks.** Every action taken by the modeler is validated against the current state of the model. If any inconsistency is found, feedback is provided to the user. What makes MSMB unique in this respect is that the feedback is intended to inform without disrupting the creative flow of model development. The modeler chooses when to fix the inconsistency. MSMB consistency checks interact with the user primarily in two ways: (1) Within MSMB’s spreadsheet-like interface, cells that contain problems are marked with a specific color to indicate the severity of the error. For example, if the user writes an expression involving a parameter called *k1* that is not yet defined, the expression cell will be colored to indicate that its definition is incomplete, but the user can still decide to go on and define other elements of the model without correcting the problem. (2) Detailed error messages that explain all outstanding errors and warnings in the model state are collected in a list and they can easily be reached by the user (Figure 2).

##### 

**Flexible autocompletion behavior.** Since a model is composed of many connected entities, MSMB can help the user by filling related tables with customizable default values. For example, if the modeler defines a reaction like “-> A”, then s/he is implicitly saying that a species A should exist in the model. If A does not exist, the tool can define it (in the Species table) with default initial conditions, relieving the user of the burden of defining all the entities before using them. This autocompletion behavior is not considered appropriate by everyone, since some users prefer to define species themselves, to avoid typographical errors. MSMB gives each user the freedom to decide the preferred behavior in these situations: (1) switch off the autocompletion completely (and have the feedback mechanism indicate a “major issue mistake” that must be fixed in the future), (2) have a pop-up window that asks for a confirmation before automatically defining any other connected entity, or (3) do autocompletion silently in the background (allowing for future correction of typos by making it easy to rename an entity).

##### 

**Content assistance autocompletion.** Complex mathematical expressions can define species or global quantities, but writing complex expressions in a model containing many entities/functions can be difficult and error prone. When filling specific cells of the spreadsheet, MSMB allows the user to trigger a drop-down menu listing all meaningful (i.e., context-dependent) entities that can be used in that mathematical expression.

Detailed examples of these autocompletion behaviors, together with descriptions of other special features of the tool, can be found in the MSMB User Manual (available at http://www.copasi.org/SoftwareProjects).

##### 

**Figure 3:**
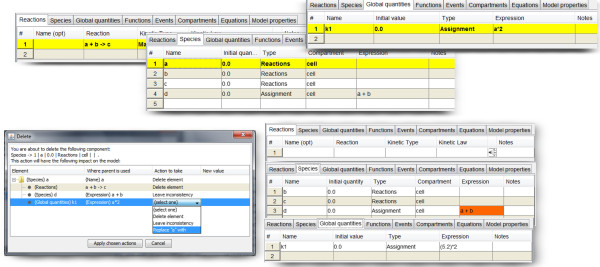
**Support with model changes: deletion of an element.** MSMB helps the user during a delete operation. The tables at the top show the initial state of the model. If the modeler decides to delete species *a*, the pop-up window shown at the bottom left corner appears. This window offers the user the choice of deleting the element, assigning a new value, or leaving the inconsistency. The bottom right part of the figure shows the state of the model after the choice of 1) deleting the reaction, 2) leaving the inconsistency in the expression of species *d*, and 3) replacing *a* with a numerical value in the expression of the global quantity *k1*.

**Rename and delete support.** Deleting a model element can have major consequences. For example, deleting a species might leave dangling references in reactions and expressions. MSMB walks the user through the problematic areas, and different options are provided to address issues that typically arise. The modeler is not forced to delete all elements that point to the one being deleted. A popup window offers the choice of deleting the element, assigning a new value, or leaving the inconsistency. Existing tools use only the delete-all option or the cannot-delete-something-that-is-used option, which restricts the modeler’s ability to follow his/her own path in maintaining the model. Limited options are good from the point of view of the tool (because they maintain the model in a consistent state at all times) but make the user’s life much harder because the user must adapt his/her actions to the tool (instead of the other way around). A similar approach is used when renaming an entity. MSMB offers flexibility (with pop-up notices that allow the user to take a different action in each case) (Figure 3). All options are available from the “Preferences” menu, and the modeler can decide to turn them on or off on a case by case basis.

##### 

**Import/export capabilities.** MSMB supports import from SBML and COPASI formats. The model can then be modified (with the possible addition of multistate species and reactions) and exported (with the proper expansion of multistate species and reactions) to SBML (level 3 version 1), COPASI, or XPP. The import/export capabilities of MSMB have been tested successfully with the 490 curated models available in the 26th release of the Biomodels database [11] and the 1196 models of the SBML Test Suite (http://sbml.org/Facilities/Database/).

### MSMB multistate syntax

In this section we describe a language to concisely encode multistate models. As briefly introduced before, multistate species are entities with a defined set of variables (called sites), each associated with an ordered list of states that describes different forms of the same conceptual species. Multistate species are involved in multistate reactions that represent a collection of reactions derived from the same kinetic law on different states of the species. A compact syntax with operators (like successor/predecessor) on the site’s states describing batches of similar reactions allows the user to define complex models in a simple, intuitive fashion.

**Figure 4:**
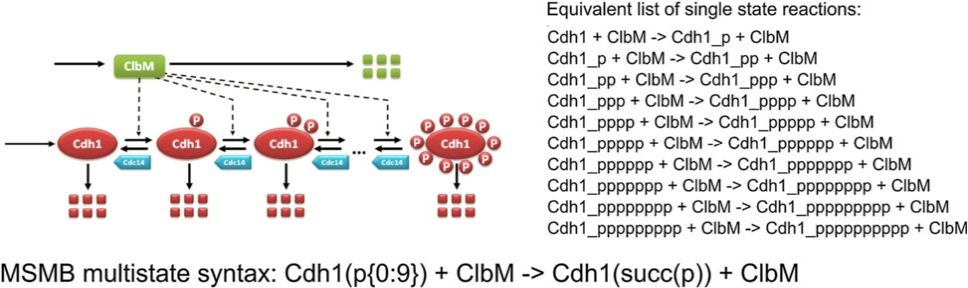
**Example of multistate species and reactions.** Protein Cdh1 has eleven forms (or states): a non-phosphorylated form (Cdh1) and ten phosphorylated forms. These are shown as a Cdh1 molecule surrounded by 1, 2,..., 10 phosphate groups (P). Each form of the protein takes part in specific phosphorylation/dephosphorylation reactions. Intuitively, the form with *i* phosphate groups has a successor form *i*+1 and a predecessor form *i*−1, with the exceptions that *i* = 0 does not have a predecessor and *i* = 10 does not have a successor. The classical way of modeling this system of phosphorylation and dephosphorylation reactions is to explicitly write the 20 single-state reactions. With our syntax the user writes just two collective reactions using successor/predecessor operators with ranges covering only the needed subset of states. In the figure, the single reaction for “successor” is shown together with the expanded form of the group of reactions modeling the phosphorylation action of Cdh1 by ClbM.

For example, as shown in Figure 4, a protein named Cdh1 is phosphorylated in each state by another protein named ClbM with the same rate law. In MSMB we can express the collection of phosphorylation reactions as a single multistate reaction.

In the following sections we present aspects of the syntax using specific models. In each example we highlight only the aspects of the syntax that are different from the previous examples, and many biological details of the model are omitted. The relevant literature is referenced, and versions of all complete models are available for download in the Examples package of the MSMB distribution.

#### Multisite phosphorylation

This section introduces the basic elements of MSMB’s multistate syntax. We illustrate the syntax with a recently published model of the budding yeast cell cycle based on multisite phosphorylation [10]. Barik *et al.* built a model of the cell cycle control network based only on mass-action kinetics (as a preliminary step to stochastic simulation). In this model, chains of multisite phosphorylation reactions generate the nonlinearities needed for the cell cycle machinery to operate correctly. Our concern here is to show how MSMB’s multistate syntax will reduce the number of reactions and species needed to represent the model.

##### 

**Basic species definition.** The main elements of a multistate species definition are the **name**, the **set of sites** and, for each of them, an **ordered list of states**. In our example, we want to model Cdh1’s states of phosphorylation. We define a species *Cdh1* with a single site (called *p*) whose states are integer values from 0 to 10. In MSMB syntax this can be written as

(3) Cdh1(p{0:10}).

MSMB language syntax uses the colon as a range operator over integer numbers. It is also possible to list each distinct state separated by a comma, allowing for the definition of sites with noninteger states or nonconsecutive integer states such as s1 {free,bound,hidden } or s2 {2,4,6,8,0 }. Allowing nonconsecutive states (as for the site *s2*) allows to compactly encode cases of multiple phosphorylation events happening in a single step, e.g., with the definition above we have that succ(2)=4. The language does not entail any meaning to the states’ values, so the interpretation of the state change succ(8)=0 is up to the modeler. If the value is an integer, MSMB allows the modeler to pass this numerical value to kinetic functions (see Section ‘Multiple phosphorylation: regulatory proteins’ for more details). MSMB also introduces the possibility of a circular list of states, connecting the last element of the list to the first for successor/predecessor operations. The circular flag available in our syntax is not used in the examples presented in this paper.

##### 

**Basic reaction definition.** The key idea of a multistate reaction is that it encodes one reaction parametrized by a collection of states from a multistate species. For example, as shown in Figure 4, Cdh1 is phosphorylated in each state by ClbM with the same rate law. In MSMB we can express the collection of phosphorylation reactions as

(4) Cdh1(p{0:9}) + ClbM -> Cdh1(succ(p)) + ClbM.

This single reaction is expanded by the tool to account for the ten single-state reactions that move the state of *p* from each single value in the range specified in the reactant (0, 1, …, 9) to its “successor” state (1, 2, …, 10, respectively). We note three important facts about the multistate reaction shown above. 

1. “succ” is a keyword that represents the successor operator. The other available operator is “pred” (for the predecessor).

2. Operators work together with the species definition. Since the species was defined as Cdh1(p {0:10 }), the successor of 0 is 1, of 1 is 2, etc. There is no implied order between the different reactions, and operators work also on noninteger states. For example with a species defined as Species(s1 {free,bound,hidden }), the reaction Species(s1 {*free*}) -> Species(succ(s1)) will be expanded as Species(s1 {*free*}) -> Species(s1 {*bound*}) because the successor of the state “free” is the state “bound” in the entity’s definition.

3. MSMB performs consistency checks to make sure that reactions are consistent with the species definition. Using Cdh1(p {0:10 }) as a reactant of the above reaction would cause an error because the successor of state 10 is not defined for species Cdh1(p {0:10 }).

##### 

**Aggregated quantities.** A species can be in different states, and many of those states can collectively affect other elements of the model (e.g., different phosphorylation states of a protein may all influence the speed of a degradation reaction of another protein). For such cases, we provide the “SUM” operator,

(5) SUM(Cdh1;p{1:10}).

“SUM” can be used in any mathematical expression to represent the sum of the concentrations of the species in the different states. A common use for this operator is to define an element (for example called Cdh1T) which represents the total amount of phosphorylated Cdh1, as shown in (5). Then Cdh1T can be used in a kinetic law as a parameter that controls the degradation of another element in the model (ClbM in this specific model). For more details about the specifics of the model, we refer the reader to model *Barik2010.msmb* included in the Examples package of MSMB.

The “SUM” operator takes the name of the species as the first argument (Cdh1 in this case), followed by a semicolon-separated list of the site restrictions that the summation should range over. In the example above, we want to sum only over the states of *p* between 1 and 10. There are many variations on the SUM operator. For example, *SUM(Cdh1)* will sum all the states, with no restrictions. The user can associate a weight function with each term of the summation, and the weight function can depend on the state value or other parameters in the model. For more examples, we refer the reader to the MSMB User Manual.

##### 

**Variable indexes.** In order to make models more flexible and easy to maintain and test, we allow ranges to be expressed using variables instead of integer constants. The disadvantage of hard-coded numbers is that if they change at any point over the lifecycle of the model, changes to all of the associated reactions/expressions are required to make sure that everything is consistent with the new values. MSMB will automatically generate a list of errors and inconsistencies, but making the changes themselves is the modeler’s responsibility. We provide an easy way of expressing ranges through external variables, called Global Quantities. In this way the change of their numerical values will be carried out seamlessly by the tool. A species definition that uses variable ranges would look like

(6) Cdh1(p{low:high}).

A reaction could look like

(7) Cdh1(p{low:high-1}) -> Cdh1(succ(p)).

Both definitions assume that “low” and “high” are defined in the model as global quantities. The idea behind this approach is that, for example, the “succ” operator usually ranges from the first state of the site definition to the penultimate state. Using the variable instead of hard-coded numbers (ten and nine in the examples) allows the modeler to test the effect of different phosphorylation chains, only changing the value of the “high” variable. MSMB will expand the entire model automatically with the new ranges.

Note that all variables used in the ranges must be statically computable. That is, their initial expression should be either a number or an expression involving numbers and/or other fixed global quantities. If the initial expression computes to a noninteger value, it will be rounded down when used in the contexts shown above.

Variables used in ranges in reactions and expressions do not necessarily have to be the same as in the species definition. For example, a modeler can use a different variable “middle” to restrict the range of the SUM in

(8) SUM(Cdh1;p{low:middle}).

Standard consistency checks will make sure that the range expressed with variables is coherent with the species definition at all times, and meaningful error messages would be shown if that is not the case.

##### 

**Figure 5:**
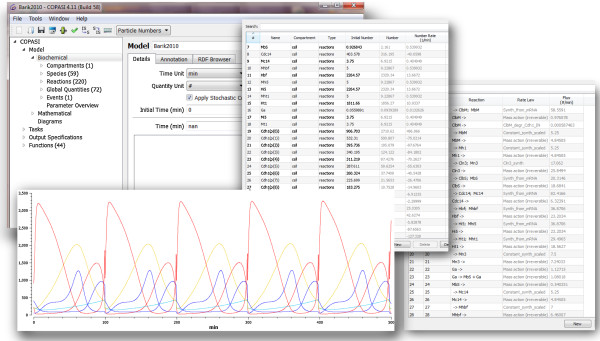
**Multisite phosphorylation model, exported to COPASI.** Parts of the Species and Reactions table are shown, together with a deterministic simulation of the model to 500 minutes.

**Exported model and simulation results.** Applying the concepts explained above on all elements of the model in [10], we can build the MSMB version of the multistate phosphorylation model of the buddying yeast cell cycle. The model file (called *Barik2010.msmb*) is included in the MSMB package. Figure 5 shows the result of exporting the model to COPASI and running a simulation for 500 minutes.

The version of the model using MSMB multistate syntax contains 23 species and 73 reactions, while the expanded single-state reaction model contains 59 species and 220 reactions. This shows that our syntax allows for a reduction in the size of the model to about one third of the original size.

##### 

**Multistate builder window.** A popup window in MSMB helps the user unfamiliar with our multistate syntax to define a multistate species. All of the rules about the site’s definition and format are laid out in the user interface, allowing the user to interactively work through the definition of the multistate species using textfields with autocompletion, dropdown menus, and radio buttons. In this way the user can become familiar with the general syntax, and will be more confident in using the more complex constructs of our grammar.

**Figure 6:**
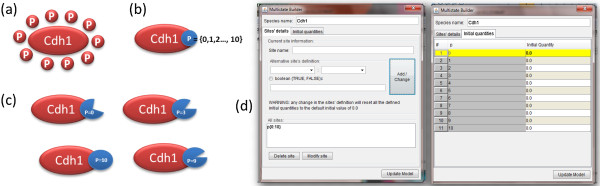
**The multistate builder window, used to modify a multistate species.(a)** Iconographic representation of a protein (called Cdh1) with ten phosphorylation sites, **(b)** Representation closer to MSMB’s encoding of the protein in (a): the species contains a single site (called P) with values ranging from 0 to 10; **(c)** Cdh1 protein can exist in several single states (i.e. specific values of the site P). The shape of the site represents the fact that different states can have different activity levels; **(d)** The multistate builder window that is used in MSMB to modify a multistate species. Information about the protein depicted in (a-c) is displayed. The interface devoted to modifying the sites structure is shown on the left. Current sites are listed in the lower part of the window. The user can select a site to modify, and its current state values will be loaded into the central part of the window. Once the changes are complete, the user can click the “Add/Change” button and the updated site will be listed. On the right, the interface devoted to the assignment of initial values for each state of the species is shown. If a species contains more than one site, all combinations of the different states of each site are generated and displayed, each with the initial quantity set as the default value for the species initial quantity. The user can specify a different initial value for the desired states. The “Update model” button saves all changes made to the model.

In the “Multistate builder” window the user can also specify the initial value of each single different state of the multistate species (Figure 6, right). The “Multistate builder” window lets the user change the name of a site and have that change propagated by the tool to all instances where the species is used.

#### Multiple phosphorylation: regulatory proteins

Kapuy et al. [12] analyzed how different phosphorylation mechanisms can influence the general properties of a model. They note that phosphorylation on multiple sites can occur progressively (i.e., during a single binding event of kinase to the substrate) or distributively (the kinase dissociates from its substrate after each phosphorylation reaction). In the latter case it may be important to consider two further options: whether the sites are phosphorylated in a specific order or the events can happen in a disordered manner.

Kapuy et al.[12] study how the properties of a distributive multistate reaction like

(9) XP(i{0:N-1}) + Kin -> XP(succ(i)) + Kin

change according to different rate law assumptions (ordered or disordered). The reaction above assumes that a species (called XP) has different phosphorylation sites (N in total) and that the number of phosphorylation sites is stored in the site called *i*. The phosphorylation reaction (coded with the “succ” operator) is carried out by a kinase (called Kin). Given the assumption of the model (i.e., that the activities are independent of the phosphorylation state of the substrate species XP) in the ordered case, the rate is the same for each step of the chain, so a simple mass-action rate law with single rate *k* is enough to encode the ordered distributive model. For the disordered mechanism, the rates of phosphorylation depend on the number of unphosphorylated sites of XP, which means that the general rate law can be expressed as (N-i) ·*k*·XP(i). This second case can be easily expressed in MSMB, using a user defined rate law (instead of mass action) to express a function (called “dis” in Table 1) that takes as arguments the total number of sites (N), the current state value (i), the kinetic constant (k) and the concentration of the reactants (XP and Kin). The definition of the two cases in MSMB format is shown in Table 1. To switch between the two cases, the modeler needs to only change the rate law of the reaction.

**Table 1:** Ordered (1) and disordered (2) reaction mechanisms for multistate phosphorylation

**Reaction**	**Type**	**Rate law**
1. XP(i {0:N-1 }) + Kin -> XP(succ(i)) + Kin	MA	k
2. XP(i {0:N-1 }) + Kin -> XP(succ(i)) + Kin	UD	dis(N,XP.i,k,XP,Kin)
**Function definition**	**Equation**	
dis(GLQ N, SITE i, GLQ k, SUB X, SUB K)	(N-i) * k * X * K	

Case 2 depends on the number of unphosphorylated states, so its rate law has to be expressed using a user defined (UD) type (instead of using mass action, MA) with a rate law function, called “dis”. The function definition is also shown. Each formal parameter has a different type (GLQ for global quantity, SITE to refer to the numerical state value of a site of a multistate species, and SUB for the substrate of the current reaction).

Kapuy et al. [12] mention that a progressive phosphorylation mechanism is also possible. In the current version of our multistate syntax it is not directly possible to encode the progressive mechanism because only the *succ*/*pred* operators are available to the user, which is sufficient support for the distributive case. However, we are planning an extension of the operators that will allow modelers to define their own *next* function, thereby encoding any possible “jump” between different states of a site.

#### Eukaryotic mRNA translation machinery

This section presents advanced features of the MSMB multistate syntax that allow modelers to deal with complexes of proteins and to express rate laws depending on groups of multistate species. For an example we use a model of the eukaryotic mRNA translation machinery. Firczuck, et al. [13] present a model that represents a 20 codon mRNA string on which ribosomes move to perform their translation task. The modelers chose this mRNA length because it allows certain key properties of translation — the effects of the physical size of the ribosome on the accessibility of the start codon and the potential for “queuing effect” along the string — to be modeled. We show how all these concepts can be encoded in MSMB syntax in a straightforward but flexible way. Our MSMB version allows the model to be scaled up to a more realistic 300 codon mRNA string with only a few changes to variable values. For more information about the model structure and the biological meaning of the species/reactions shown below, we refer the user to the original publication.

##### 

**Transfer of state between multistate species.** In a model, different species may need to share multistate site values. For example, the model of [13] contains six different complexes that are encoded as six separate species with names that remind the modeler what the different components in the complex are. We show how to encode complexes in MSMB in the next subsection; however, in our syntax it is possible to encode species exactly as in the original publication. The “transfer of state between multistate species” allows a smooth encoding of the ideas in the original model, with the advantage of compressing the current position of a species on the mRNA string in a multistate site, instead of using the “_number”-added-to-the-name approach of the original publication, which is not scalable nor easy to maintain.

An example of a reaction using the “transfer state” concept is

$$\begin{array}{lll} & \text{aatRNA\_eEF1A\_GTP}+\text{80S(codon)}\; & \;\\ & \;\text{-> 80S\_aatRNA\_eEF1A\_GTP(codon=80S.codon)}.\; & \; \end{array}$$

This reaction assumes that three species are defined in the model: a regular species *aatRNA_eEF1A_GTP*, a multistate species *80S* with one site *codon*, and another multistate species *80S_aatRNA_eEF1A_GTP* with one site *codon*^a^. The concept of “transfer state” can be seen in the product of the reaction, where the value of the codon site of the product is taken from the value of the codon site in the reactant. Note that even if the name of the site of the two species is the same, the “reactantName.reactantSite” format has to be used because, in general, the assignment can be done between sites with different names and/or different reactant species sharing the same site name. Standard consistency checks will make sure that the definitions of the two species are compatible and that the assignment does not refer to nonexisting states of the product.

The concept of “transfer state” can be combined with other concepts in the multistate syntax (e.g., operators and variable ranges to restrict reactants) to write more complex reactions, such as:

$$\begin{array}{lll} & \;\text{80S\_aatRNA\_eEF2\_GTP(codon\{}\mathrm{1:criticalCodon}\text{\})}\;\text{->}\; & \;\\ & \;\text{80S\_tRNA(codon}\mathrm{=succ}\text{(80S\_aatRNA\_eEF2\_GTP.codon))}\; & \;\\ & \;+\text{eEF2\_GDP}.\; & \; \end{array}$$

This reaction illustrates the fact that not only the position of the species is moved forward (succ) but also the “state” of the species is changed and encoded with a different species name. This reaction is restricted to a specific set of positions (1 to criticalCodon) because the rate of this reaction is influenced by the state of other proteins in the system that may be in following positions on the mRNA string (queueing effect). This requires the single reaction of “translocating on the mRNA string” to have three separate cases, driven by different rate laws. Our multistate syntax allows us to write the three cases in a straightforward way. We define a criticalCodon variable that represents the threshold value for these cases. The reaction above represents the first case. A reaction only happening for 80S_aatRNA_eEF2_GTP(codon {*criticalCodon*}) would be the second case. The final case would restrict the reactant to the range “criticalCodon+1:lengthmRNA”. The last case is driven by a simple mass action rate law, but the first two require more sophisticated rate laws that will be illustrated in the next section. For the motivation of those kinetic choices, we refer the reader to the original publication [13].

##### 

**Aggregate reaction modifiers.** In special cases, the rate of a reaction may be influenced by a specific subset of a multistate species. To encode this, multistate species can be defined with assignments depending on specific values of a state. To clarify this scenario we use the reaction with complex transfer state explained in the previous subsection, renaming the species as R (for 80S_aatRNA_eEF2_GTP), P1 (for 80S_tRNA) and P2 (for eEF2_GDP), the site codon as c, and the variable criticalCodon as d, yielding

(10) R(c{1:d}) -> P1(c=succ(R.c)) + P2; M(c=R.c).

We introduce a modifier (multistate) species M, whose site value c will take the value of the site c in the reactant R (i.e., each different expanded reaction will have a different value for R.c and that value will be passed down to M for further calculations). The definition of M will be a special case of multistate as M(c), with no state values for c, but defined with an assignment using the SUM operator as

(11) M(c) assigned the value SUM(R;c{M.c+1:M.c+14]}.

This SUM operation will sum the concentration of all the states of R where site c takes the value between the current M.c value plus one to the value of the current M.c value plus 14. To make the concept clearer, some of the expanded single-state reactions generated by the above definition are

$$\begin{array}{lll} & \text{R(c\{1\}) -> P1(c\{2\})}+\text{P2; M(1)},\; & \;\\ & \;M(1)=\text{R(c\{2\})}+\ldots +\text{R(c\{16\})};\; & \;\\ & \text{R(c\{2\}) -> P1(c\{3\})}+\text{P2; M(2)},\; & \;\\ & \;M(2)=\text{R(c\{3\})}+\ldots +\text{R(c\{17\})},\; & \;\\ & \ldots \; & \; \end{array}$$

Once M is introduced as a modifier of the reaction and properly defined to range over a specific subset of the reactant state values, species M can be passed as a parameter in any function, and any rate law can be expressed in terms of M as required by the model. For more details about the specifics of the model, we refer the reader to the model *Firczuck2013.msmb* included in the Examples package of MSMB.

##### 

**Figure 7:**
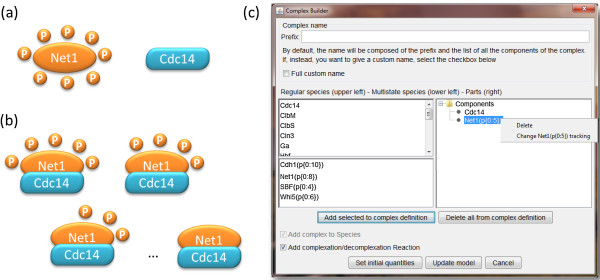
**Complex Builder wizard.(a)** Iconographic representation of two species: a multistate species (called Net1) with eight phosphorylation sites, and a simple species (called Cdc14). **(b)** Net1 and Cdc14 protein form a complex only with a limited number of states of Net1 (i.e. not all possible phosphorylation states of Net1 can form a complex with Cdc14). In this case, only states where between 0 and 5 phosphorylation sites allow the complex formation. **(c)** Complex Builder wizard, the window that helps the user manage complex formation with multistate species. The window is divided into three parts: top, bottom, and central. The top is where the name of the complex may be specified, following the rules of generic species names, but the wizard allows the user to have the name picked (and maintained) by MSMB so that it reflects the components that are listed in the complex. At the bottom, the user can decide to add only the species complex to the model (default) or add as well the complexation and decomplexation reactions in the list of reactions. Those reactions have an elaborate syntax (involving the “transfer state” concept) so the user can let the tool handle their creation. The center right area is where the current composition of the complex is displayed. Each component can be deleted or, if of multistate kind, its tracking can be changed. The center left area is a list of available species of the model that can be added as components of the complex. Regular species are listed in the upper list, multistate species are listed in the lower list.

**Complex builder window.** MSMB has also the notion of a complex, which is a species composed of other species connected to form a single unit. The complex can be used in any context in which a regular species appears, but it has the advantage that if one of its components is changed by the user (e.g., renamed) those changes are seamlessly carried to all the complexes that contain that (now modified) species. MSMB does not track each individual bond formation/breakage, but we help the user manage complex formation/breakage through the “Complex Builder” window (Figure 7).

The “Complex Builder” window is similar to the “Multistate Builder” window, as it can be used to specify the initial condition of different states of the complex and it must be used to make changes to its components. This interactive way of building complexes offers the user the chance to specify the complex with a few clicks and let MSMB deal with the more complicated issues (like transfer state for complexation and decomplexation reactions, renaming of species components, change in the components’ multistate structure, etc).

##### 

**Figure 8:**
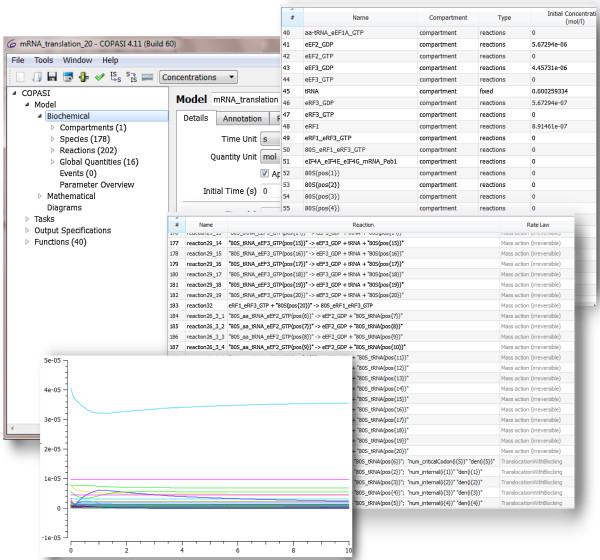
**mRNA transcription model, exported to COPASI.** Parts of the Species and Reactions table are shown, together with a deterministic simulation of the model to 10 seconds.

**Exported model and simulation results.** Applying the concepts explained above on all elements of the model in [13], we build the MSMB model of the mRNA translation machinery. The model file *Firczuck2013.msmb* is included in the Examples package of MSMB. Figure 8 shows the result of exporting the model to COPASI and running a simulation for 10 seconds.

The model using MSMB multistate syntax contains 60 species and 58 reactions, while the expanded single-state model contains 178 species and 202 reactions, a reduction in the size of the model to less than one third of the original size. Since we use the variable range concept to encode the model with 20 codons, changing the model to one that contains 300 codons requires only a change of a single numerical variable and MSMB, at export, will expand it to 2162 reactions and 2418 species. Testing different hypotheses about the size of ribosome occupancy (encoded in the model as the variable called “criticalCodon”) would require only a change in its numerical value, and MSMB would generate the new model for further testing.

## Conclusions

We have implemented a new flexible model editor that helps users write complex biochemical reaction models in a compact way. MSMB offers extensive user customization and functionalities that facilitate model creation (e.g., autosaving, autocompletion, compact printout of the entire model, full customization of the tool default values, customization of tool’s behavior with complex model changes like renaming/deleting elements). MSMB offers import/export functionality to/from SBML and COPASI, allowing a smooth integration of MSMB with any existing simulation/analysis tools that use these standard formats.

MSMB introduces a new compact and powerful syntax for multistate species, which have not been fully supported by many existing SBML-related tools. The importance of the multistate concept is also acknowledged by the SBML community through the ongoing effort of defining a *multi* package for the SBML Level 3 specification (http://sbml.org/Community/Wiki/SBML_Level_3_Proposals/Multistate_and_Multicomponent_Species). The *multi* package’s purpose is to define “object structures for representing entity pools with multiple states and composed of multiple components, and reaction rules involving them”, toward which the proposed compact multistate syntax contributes.

MSMB has been tested on different models developed by Tyson’s group and other published multistate models, as well as on all models in the Biomodels database and in the SBML Test Suite. In the future, we plan to implement user-defined operators on states and more aggregated quantities functions, as well as more features to improve the user experience and productivity.

## Availability and requirements

1. **Project name:** MSMB

2. **Project home page:**http://www.copasi.org/SoftwareProjects

3. **Operating system(s)**: Platform independent

4. **Programming language:** Java

5. **Other requirements:** Java 6.0 or higher

6. **License:** Artistic License 2.0

## Endnote

^a^ Note that the names of the species are quite elaborate, but for MSMB they don’t have specific meaning. It is the modeler’s decision to use intricate names that may help the readability of the model in terms of its biological interpretation. The need for intricate species names motivated the implementation of MSMB’s name autocompletion mechanism.

## Competing interests

The authors declare that they have no competing interests.

## Authors’ contributions

AP implemented the source code and drafted the manuscript. CAS, LTW, SH and JJT initiated and coordinated the project. All authors participated in extensive discussions leading to the definition of the multistate syntax. All authors participated in and approved the final manuscript’s preparation.
